# A Comparative Analysis of E-Cigarette and Cigarette Posts on Instagram

**DOI:** 10.3390/ijerph20043116

**Published:** 2023-02-10

**Authors:** Young-shin Lim, Ji Young Lee

**Affiliations:** 1 Graduate School of Business, Sejong University, Seoul 05006, Republic of Korea; 2 Independent Researcher

**Keywords:** e-cigarette, cigarette, public health, social media, Instagram, content analysis

## Abstract

E-cigarette use has grown rapidly over the past decade and become a threat to public health. Marketing—especially through social media—has contributed significantly to this growth, which suggests that regulating content in social media will be critical in supporting efforts to reverse this trend. A content analysis was performed to compare 254 e-cigarette posts on Instagram with 228 cigarette posts on the same platform. The majority of e-cigarette posts were from e-cigarette companies (40.9%) and industry people (18.5%), whereas the majority of cigarette posts were from laypeople (76.8%). More e-cigarette posts than cigarette posts appeared to have a marketing intent (56.3% vs. 1.3%), and brand representation in photographs/videos was more frequent in the e-cigarette posts than in the cigarette posts (63.0% vs. 15.8%). Further, compared with the e-cigarette posts, the cigarette posts were more likely to portray daily life (73.2% vs. 41.3%) and humans (80.3% vs. 43.7%) in the photograph/video. The cigarette posts also portrayed smoking much more often than the e-cigarette posts portrayed vaping (67.1% vs. 21.3%). The study findings broaden the field’s understanding of cigarette and e-cigarette content on Instagram and social media, and have implications for monitoring and regulating content for e-cigarettes and cigarettes.

## 1. Introduction

Electronic cigarettes (e-cigarettes)—a type of electronic nicotine delivery system (ENDS)—constitute a growing public health concern [1]. Unlike conventional, combustible cigarettes, which burn tobacco, with e-cigarette devices, users take in an aerosol generated by heating a liquid solution (referred to as e-liquid or e-juice) [1]. E-cigarettes are also called “e-cigs”, “e-hookahs”, “mods”, “vape pens”, “vapes”, and “tank systems”, and the act of using e-cigarettes is referred to as “vaping” [2].

E-cigarettes have often been presented as a safer alternative to cigarettes [3,4]. However, although they may not be as harmful as cigarettes, e-cigarettes are still not safe and are, therefore, associated with health risks [4,5]. Despite involving fewer toxic substances compared to cigarette smoking, e-cigarettes still contain harmful chemicals [2,6,7,8]. It should be well noted that the e-liquids used in vaping typically contain nicotine, which is known to be highly addictive and, therefore, makes quitting vaping difficult to achieve [1]. It is also the case that vaping can have a detrimental effect on adolescent brain development [9]. In addition to nicotine, the e-cigarette aerosol includes toxic substances such as heavy metals, volatile organic compounds, ultrafine particles, cancer-causing chemicals, and flavoring that may cause lung disease [2]. Previous studies document a range of harmful effects from the use of e-cigarettes, such as increased respiratory complications and increased cardiovascular risk [5]. In particular, in 2019, in the United States, there were multiple e-cigarette or vaping product use-associated lung injury (EVALI) cases related to vitamin E acetate [10]. The US Centers for Disease Control and Prevention (CDC) recorded 2807 hospitalized or death cases due to EVALI by February 2020 [10]. Considering the potential for significant ill effects on people’s health, the World Health Organization (WHO) strongly advises countries to recognize ENDS as harmful products and to regulate them accordingly [1,4].

In recent years, the e-cigarette market has experienced rapid growth; specifically, the global market size increased from 7.76 billion US dollars in 2014 to 22.82 billion US dollars in 2022, with an expected annual growth of 3.44% by 2027 [11]. According to the US CDC, in 2018, 14.9% of US adults had used e-cigarettes and 3.2% were current users [12]. Even more concerning is the extent of e-cigarette use among the country’s youth for whom e-cigarettes have been the most prevalent form of tobacco product used since 2014 [13]. From 2011 to 2019, whereas the cigarette use rate dropped from 15.8% to 5.8% among US high school students, the e-cigarette use rate of this group increased from 1.5% to 27.5% during the same period [14,15]. Due to this rapid increase, in 2018, the US Surgeon General issued an advisory declaring youth e-cigarette use an epidemic and urging action to protect young people [16]. Although youth e-cigarette use has declined somewhat since then, the 2022 National Youth Tobacco Survey indicates that the figures remain high: Approximately 2.5 million US youth currently use e-cigarettes (14.1% of high school students and 3.3% of middle school students) [17].

Marketing is one of the key drivers of e-cigarette use. In particular, the product is marketed extensively on the Internet including via social media [18,19,20]. The 2021 US National Youth Tobacco Survey indicated that approximately 74% of youth who use social media had been exposed to content related to e-cigarettes [21]. In a recent longitudinal study, Chen-Sankey and colleagues demonstrated an association between youth and young adults’ exposure to e-cigarette marketing and their future e-cigarette experimentation [22]. More specifically, based on another longitudinal study, Zheng and Lin demonstrated that the effect of youth’s exposure to online e-cigarette advertisements on their subsequent e-cigarette use is sequentially mediated through perceived social norms and risk perceptions regarding e-cigarette consumption [23]. The WHO’s recommendation to regulate ENDS encompasses regulating advertising, promotion, and sponsorship in an effort to prevent children and non-smokers from using e-cigarette products [1]. Compared to the regulation of cigarette advertising, the regulation of e-cigarette advertising is at a relatively early stage such that further investigation into ongoing advertising practices and portrayals of e-cigarettes in various kinds of media is needed [24,25]. In this regard, the purpose of the present study is to investigate how e-cigarettes (vs. conventional cigarettes) are portrayed on the popular social media platform Instagram.

Instagram is a social media platform that offers users an easy way to instantly share photographs and videos. In 2022, more than 2 billion people used the platform each month [26]. Instagram users record their everyday lives, express themselves, and interact with others on the platform [27]. According to the Pew Research Center, Instagram is one of the most popular social media platforms among younger generations, and 76% of US adults aged 18 to 24 used the platform in 2021 [28]. A majority (59%) of Instagram users said that they use the platform at least once a day [28]. Further, in 2022, Instagram was the third most popular platform among US teenagers (aged 13 to 17) with 62% stating that they use the platform, following only YouTube with 95% and TikTok with 67% [29].

In terms of e-cigarette propagation, social media serves as a conduit for social factors that may influence people’s e-cigarette use such as e-cigarette advertising, social interactions, and social norms [30]. In general, social media is an important part of contemporary marketing communication, and the visual-focused nature of Instagram makes it an effective marketing tool [27,31]. On Instagram and in relation to smoking and vaping specifically, users share their cigarette and e-cigarette consumption, including via smoking selfies, which are frequently shared on the platform [32]. Several studies report the prevalence of e-cigarette- and vaping-related posts on Instagram and other social media. A previous study reported the visually appealing characteristic of ENDS posts on Instagram and their potential effects on viewers’ perceptions of the products [33]. One noticeable aspect of e-cigarette and vaping posts on Instagram is the prevalence of advertising and promotional posts by e-cigarette businesses [34,35].

Significant differentiation is evident between cigarette brands and e-cigarette brands in relation to social media use. O’Brien and colleagues investigated the social media presence and use of leading US brands of various types of tobacco products (cigarettes, e-cigarettes, cigars, hookahs, and smokeless tobacco) [20]. The researchers found that 91.4% of the leading e-cigarette brands had a page on at least one social media platform and 80.0% had a page on three or more social media platforms. In contrast, 95.5% of the leading cigarette brands did not have a page on a social media platform. Moreover, Instagram was the most-used platform in this regard by leading e-cigarette brands: 82.9% of such brands had a page with posts on Instagram, followed by 80.0% on Facebook and likewise 80.0% on YouTube, and then 77.1% on Twitter. However, not one of the cigarette brands included in the study had a page on Instagram. A difference of this nature is likely to influence portrayals of e-cigarettes and cigarettes on Instagram. Although the literature includes studies investigating e-cigarette posts on social media [34,35,36,37], few studies offer a comparative analysis of e-cigarette posts and cigarette posts.

Given this gap in the literature, the present study investigates whether and how (1) e-cigarette posts and cigarette posts differ in terms of their respective sources (posting accounts), (2) e-cigarette posters and cigarette posters differ in regard to their account activities, (3) e-cigarette posts and cigarette posts differ in terms of marketing intent and sharing of promotional information, (4) e-cigarette posts and cigarette posts differ in terms of the media type shared (photograph or video), (5) the photographs/videos in the e-cigarette posts and the cigarette posts differ in terms of their portrayals of daily life, vaping/smoking, humans, e-cigarette/cigarette artifacts, and brands, and (6) user engagement with the posts differs between e-cigarette and cigarette. This comparison will afford a stronger and more nuanced understanding of how e-cigarette companies’ marketing efforts influence portrayals of e-cigarettes (vs. cigarettes) and promote interactions on Instagram.

## 2. Methods

A manual content analysis of Instagram posts was performed to compare e-cigarette and cigarette posts.

### 2.1. Sampling

Following the literature, sampling was based on a search of keywords relevant to e-cigarettes and cigarettes [36,38]. First, using #ecig and #cigarette with Instagram’s search function, we identified popular hashtags associated with Instagram postings related to e-cigarettes and cigarettes. Through this initial search, we identified four popular hashtags related to each kind of tobacco product: #vape, #vapor, #vaping, and #ecig for e-cigarettes and #smoke, #smoking, #cigarette, and #cigarettes for cigarettes.

The sampling process consisted of two stages. In the first stage (between 29 August 2016 and 31 August 2016), we collected the top 70 search results (i.e., 9 top posts and 61 recent posts) for each of the eight hashtags (i.e., 560 posts in total), and then we took screenshots of each post and recorded its URL. Further, for each post collected, we also took a screenshot of the poster’s profile page and recorded its URL. In addition to these 560 posts, we collected 60 posts following this same approach in order to develop the coding scheme. Second, three days after the first stage, we revisited the posts and took more screenshots to collect evidence of user engagement—i.e., the number of likes/views and comments—that the posts had accrued. The engagement data of any posts deleted before the second stage were treated as missing. Duplicated posts, posts irrelevant to e-cigarettes or cigarettes, and posts not written in English were excluded from the sample. The final data consisted of 482 posts (254 e-cigarette posts, 228 cigarette posts) by 429 unique accounts (237 e-cigarette accounts, 192 cigarette accounts).

### 2.2. Coding Process and Scheme

Two trained coders coded the sampled posts and profile pages. After a training session, approximately 11.8% of the data were used for reliability coding. After each of the two coders had independently coded the reliability coding dataset, one of the investigators and both coders reviewed all cases of disagreement and reached an agreement based on discussion. There was one more round of reliability coding with approximately 12.2% of the data for the codes that reliability was not established through the first round. After reliability had been established, the rest of the data were split and independently coded by the coders. The coding scheme and the reliability scores are discussed next.

The post sources were classified based on the account profile page and other posts on the profile page. Specifically, the source variable was coded according to six categories: layperson, cigarette/e-cigarette company, industry person, health organization/practitioner, community, and other/unidentifiable (Krippendorff’s alpha = 0.93). The layperson category refers to noncommercial, personal accounts of people not working in or sponsored by the cigarette/e-cigarette industry. The cigarette/e-cigarette company category refers to accounts officially associated with a company, brand, and/or vendor. The industry person category refers to accounts associated with a person who is either part of or sponsored by the cigarette/e-cigarette industry. E-cigarette sponsorship is documented in the literature [36,39]. We identified sponsorships by consulting acknowledgements in the profile pages. The health organization/practitioner category refers to accounts associated with health organizations as a whole such as medical networks or medical associations and accounts of individual health practitioners. The community category refers to accounts representative of or posting content from a group of people. The other/unidentifiable category refers to all accounts that do not fit any of the other designated categories.

In addition to the classification described, each account was examined to determine whether it was a themed account. The poster’s account profile page was coded dichotomously based on whether e-cigarettes or cigarettes were the primary topics of the posts from the account (Krippendorff’s alpha = 1.00).

In regard to account activities, three numbers, i.e., the number of posts, the number of followers, and the number of followings, were coded from the account profile page (Krippendorff’s alphas = 1.00, 1.00, 0.97, correspondingly). In cases of numbers indicated in “k”, the unindicated digits were treated as 0. For example, “12.7k” was coded as “12,700”.

For marketing intent, dichotomous coding was used with each post (photograph/video and text message together) coded depending on whether or not it appeared to have a marketing intent, i.e., to influence the attitudes or purchase decisions of customers or potential customers (e.g., advertisements, sponsored postings featuring e-liquid flavors) (Krippendorff’s alpha = 0.85).

More specific to marketing intent, the posts’ sharing of promotional information was examined. Dichotomous coding was used with each post coded depending on whether or not it provided specific promotional information such as sales promotions, events, and/or sample giveaways (Krippendorff’s alpha = 0.74).

The media type shared by the posts was examined. Each post was classified based on whether it shared a photograph or a video (Krippendorff’s alpha = 1.00).

The portrayal of daily life was dichotomously coded according to whether or not the photograph/video depicted daily life. Photographs/videos with content such as vape tricks, representations of everyday life, drawings, screen captures, smoking/vaping pictures, and selfies including smoking/vaping were coded as depicting daily life (Krippendorff’s alpha = 0.89).

The portrayal of smoking/vaping was dichotomously coded according to whether or not the photograph/video shared in a post shows smoking/vaping (Krippendorff’s alpha = 0.96).

The portrayal of humans was dichotomously coded according to whether or not the photograph/video depicted any human (or any part of the human body, e.g., hands) (Krippendorff’s alpha = 0.96).

The portrayal of cigarette/e-cigarette artifact was originally examined with two separate codes: the portrayal of cigarette/e-cigarette and portrayal of e-liquid. E-cigarette artifacts are more complicated than conventional cigarette artifacts as e-cigarette devices (mods) have cartridges that need to be refilled with e-liquid. The variety of e-liquid flavors available has been indicated as a reason for vaping [40]. For the portrayal of cigarette/e-cigarette, each post was dichotomously coded according to whether the photograph/video presents a cigarette (for cigarette posts) or an e-cigarette mod (for e-cigarette posts) (Krippendorff’s alpha could not be calculated for this code; there was a 100% agreement with no variation in the reliability dataset). For the portrayal of e-liquid, each post was dichotomously coded according to whether or not the photograph/video shows e-liquid (Krippendorff’s alpha = 0.86). However, later in the coding process, the two codes were collapsed and recoded according to whether the photograph/video presents a cigarette/e-cigarette mod and/or e-liquid.

The portrayal of a brand was dichotomously coded based on whether the photograph/video presented a brand related to cigarettes or a brand related to e-cigarettes (Krippendorff’s alpha = 0.86).

User engagement was coded based on the screen captures of the posts three days after the original sampling. On Instagram, users can “like” or “comment” on a post, and the number of likes and the number of comments show up with the post. For a post that shares a video, the number of “views” show up when the post appears as a search result. For all posts, the number of comments was coded (Krippendorff’s alpha = 0.97). For posts sharing a photograph, the number of likes was coded (Krippendorff’s alpha = 0.97). For posts sharing a video, the number of views was coded (Krippendorff’s alpha = 1.00). For the account activity codes, when the numbers were indicated in “k”, the unindicated digits were treated as 0.

## 3. Results

### 3.1. Sources

A 2 × 6 chi-square analysis was performed to determine any differences between the cigarette posts and the e-cigarette posts in terms of the ratio of posts for each source type. The chi-square revealed a significant difference between cigarette posts and e-cigarette posts regarding the source classification, *χ*^2^(5, *N* = 482) = 192.78, *p* < 0.001. E-cigarette companies were the most frequent source of e-cigarette posts (40.9%), whereas laypeople were the most frequent source of cigarette posts (76.8%). Table 1 presents the ratio of posts by source type.

Moreover, the posts were examined to determine whether they were posted by a themed account—an account with a principal focus on cigarettes or a principal focus on e-cigarettes. A chi-square analysis revealed that e-cigarette posts were significantly more likely to be from accounts that primarily post about this topic (89.4%) as compared with cigarette posts (21.5%), *χ*^2^(1, *N* = 482) = 226.21, *p* < 0.001.

In addition to the ratio of posts by source, the source accounts were investigated based on data comprising 429 unique accounts (55.2% e-cigarette accounts, 44.8% cigarette accounts). In accordance with the above results, the classifications of the cigarette posters and the e-cigarette posters were significantly different, *χ*^2^(5, *N* = 429) = 187.74, *p* < 0.001. Table 2 presents the classification of the sources. Moreover, 88.6% of the e-cigarette posters showed a primary focus on the topic of e-cigarettes, whereas only 8.9% of the cigarette posters showed a primary focus on the topic of cigarettes, *χ*^2^(1, *N* = 429) = 270.79, *p* < 0.001.

### 3.2. Account Activities

Three sets of one-way ANOVAs were performed to examine differences in the account activities of the cigarette posters and the e-cigarette posters. An ANOVA indicated that the e-cigarette posters (*M* = 754.09, *SD* = 1456.72, *Median* = 254.00) had significantly more accumulated posts as compared to the cigarette posters (*M* = 454.79, *SD* = 884.75, *Median* = 140.50), *F*(1, 427) = 6.24, *p* = 0.013, partial *η*^2^ = 0.014). In addition, the e-cigarette posters (*M* = 16,300.57, *SD* = 47,865.35, *Median* = 1352.00) had more followers than the cigarette posters (*M* = 3107.45, *SD* = 15,313.63, *Median* = 309.50), *F*(1, 427) = 13.46, *p* < 0.001, partial *η*^2^ = 0.031). Lastly, the e-cigarette posters were following more accounts (*M* = 1118.41, *SD* = 1618.13, *Median* = 488.00) than were the cigarette posters (*M* = 598.09, *SD* = 1205.71, *Median* = 249.00), *F*(1, 427) = 13.69, *p* < 0.001, partial *η*^2^ = 0.031). Table 3 summarizes the account activities according to source type.

### 3.3. Marketing Intent and Promotional Information

A chi-square analysis revealed a significant difference in the presumed marketing intent of the cigarette and e-cigarette posts, *χ*^2^(1, *N* = 482) = 172.02, *p* < 0.001. Approximately 56.3% of the e-cigarette posts were presumed to have a marketing intent, whereas only 1.3% of the cigarette posts were presumed to have a marketing intent.

A chi-square analysis indicated a significant difference in sharing of promotional information between the cigarette posts and the e-cigarette posts, *χ*^2^(1, *N* = 482) = 33.88, *p* < 0.001. Approximately 13.8% of the e-cigarette posts shared specific promotional information, whereas not one cigarette post shared this kind of information.

### 3.4. Media Type

A chi-square analysis revealed a significant difference between the cigarette posts and e-cigarette posts in terms of the type of media (photograph or video) shared, *χ*^2^(1, *N* = 482) = 22.18, *p* < 0.001. Overall, 6.4% of the posts shared a video. Only 0.9% of the cigarette posts shared a video, compared with 11.4% of the e-cigarette posts sharing content in this form.

### 3.5. Portrayals in Photographs/Videos

A chi-square analysis revealed a significant difference between the e-cigarette posts and the cigarette posts in terms of the portrayal of daily life, *χ*^2^(1, *N* = 482) = 49.75, *p* < 0.001. The cigarette posts were more likely to share a photograph/video depicting daily life (73.2%) than were the e-cigarette posts (41.3%).

A chi-square analysis indicated a significant difference regarding the portrayal of smoking/vaping in the photographs/videos between the cigarette posts and the e-cigarette posts, *χ*^2^(1, *N* = 482) = 103.06, *p* < 0.001. The cigarette posts were more than three times as likely to portray smoking (67.1%) than the e-cigarette posts were to portray vaping (21.3%).

A chi-square analysis revealed a significant difference regarding human portrayal in the photographs/videos between the cigarette posts and the e-cigarette posts, *χ*^2^(1, *N* = 482) = 67.51, *p* < 0.001. The cigarette posts were close to twice as likely to portray humans (80.3%) compared to the e-cigarette posts (43.7%).

A chi-square analysis indicated a significant difference in portrayals of cigarette/e-cigarette artifacts in photographs/videos between the cigarette posts and the e-cigarette posts, *χ*^2^(1, *N* = 482) = 9.51, *p* = 0.002. Approximately 97.8% of the cigarette posts portrayed cigarettes. Approximately 91.3% of the e-cigarette posts portrayed e-cigarette artifacts (63.0% presented an e-cigarette device, 42.1% presented e-liquid, and 13.8% presented both).

A chi-square analysis revealed a significant difference in the portrayal of brands in the photographs/videos of the cigarette and e-cigarette posts, *χ*^2^(1, *N* = 482) = 110.95, *p* < 0.001. The e-cigarette posts were almost four times more likely to portray a specific brand in the photographs/videos (63.0%) than were the cigarette posts (15.8%).

### 3.6. User Engagements

A one-way ANOVA was performed to test the difference in the number of likes between the cigarette posts and the e-cigarette posts. The ANOVA indicated no significant difference in the number of likes, *F*(1, 431) = 1.51, *p* = 0.220, partial *η*^2^ = 0.003, accrued by the cigarette posts (*M* = 156.24, *SD* = 580.66, *Median* = 31.00) and the e-cigarette posts (*M* = 254.48, *SD* = 1016.70, *Median* = 68.50).

As e-cigarette posts accounted for the vast majority of posts sharing a video (29 e-cigarette posts; two cigarette posts), we simply performed descriptive analyses for the number of views instead of undertaking an inferential analysis. For the e-cigarette posts, the mean number of views was 7990.97 (*SD* = 16,943.87, *Median* = 178.00). For the cigarette posts, the mean number of views was 114.00 (*SD* = 65.05, *Median* = 114.00).

A one-way ANOVA was performed to compare the number of comments made on the cigarette posts and the e-cigarette posts. The ANOVA indicated a significant difference in the number of comments on the two types of posts, *F*(1, 462) = 6.10, *p* = 0.014, partial *η*^2^ = 0.013. The e-cigarette posts accrued more comments (*M* = 6.66, *SD* = 16.78, *Median* = 2.00) than did the cigarette posts (*M* = 3.20, *SD* = 12.84, *Median* = 1.00).

### 3.7. Summary of Results

Overall, the findings suggest differences in the composition of the e-cigarette posters and cigarette posters, and accordingly, e-cigarette posts and cigarette posts were found to differ in several aspects such as marketing intent, media type, portrayals in the photograph/video content, and user engagement. The majority of e-cigarette posts were posted by e-cigarette companies (40.9%) and industry people (18.5%), whereas the majority of cigarette posts were from laypeople (76.8%). Posts by laypeople accounted for 27.2% of e-cigarette posts, and posts by cigarette companies accounted for only 1.3% of the cigarette posts. The majority of the e-cigarette posts were from themed accounts that primarily post about e-cigarettes (89.4%), whereas 21.5% of the cigarette posts were from accounts that primarily post about cigarettes. The accounts posting about e-cigarettes were more active than the accounts posting about cigarettes in terms of the number of accumulated posts, the number of followers, and the number of followings. More e-cigarette posts than cigarette posts were presumed to have marketing intent (56.3% vs. 1.3%), and likewise, more e-cigarette posts than cigarette posts were found to share specific promotional information (13.8% vs. 0%). Videos were shared more frequently in e-cigarette posts than in cigarette posts (11.4% vs. 0.9%). Brand portrayals in photograph/video content were more frequent in e-cigarette posts than in cigarette posts (63.0% vs. 15.8%). On the other hand, in the photograph/video content, compared to e-cigarette posts, cigarette posts were more likely to portray daily life (73.2% vs. 41.3%) and humans (80.3% vs. 43.7%). In addition, the cigarette posts were also more likely to show smoking than the e-cigarette posts were to show vaping (67.1% vs. 21.3%) and more likely to show cigarette artifacts than the e-cigarette posts were to show e-cigarette artifacts (97.8% vs. 91.3%). Lastly, the e-cigarette posts accrued more comments than did the cigarette posts (6.66 vs. 3.20), although there was no significant difference in the number of likes accrued. The results have implications for both cigarette and e-cigarette portrayals on Instagram.

## 4. Discussion

The e-cigarette market has grown rapidly over the past decade [11]. In particular, there has been dramatic growth in e-cigarette use among youth such that in 2018 the US Surgeon General declared youth e-cigarette use to be an epidemic [16]. In fact, it has become evident that the use of e-cigarettes presents a serious threat to public health considering their potential to cause respiratory and cardiovascular disease, harm to adolescent brain development, and even, death [5,9,10]. Furthermore, even with these potentially harmful consequences for users, it remains the case that very little is known about the long-term effects of e-cigarette consumption [5].

Marketing, including and possibly principally through social media, has contributed greatly to the rapid growth of e-cigarette use. In fact, the e-cigarette industry’s marketing efforts have focused to a large extent on using social media platforms popular among youth [41]. In contrast to restrictions placed on the marketing of traditional tobacco products, e-cigarette marketing was not subject to similar restrictions until recently [41]. Further, given the harm e-cigarettes can cause, the WHO is urging countries to regulate the advertising, promotion, and sponsorship of ENDS products [1]. In 2021, the U.S. Food and Drug Administration required information from four e-cigarette brands about their social media marketing practices [42]. Efforts to regulate the availability and marketing of e-cigarettes remain at an early stage of development with a stronger understanding of current practices needed to inform this direction [24,25]. Further, social media is evidently a primary marketing conduit for e-cigarette companies. For these reasons, our focus in the present study is on analyzing e-cigarette and cigarette posts on Instagram, where e-cigarette brands are actively present and where cigarette brands have only a limited presence [20]. While there have been studies that investigated e-cigarette posts on social media [34,35,36,37], there has been limited focus on comparing e-cigarette posts with cigarette posts. The present study aimed to fill in such a gap in the literature.

First, corresponding to the literature [36,37], e-cigarette companies and industry people were responsible for most of the e-cigarette posts (59.4% together). On the other hand, most of the cigarette posts were from laypeople (76.8%), everyday individuals with no apparent affiliation with the industry. Moreover, most of the e-cigarette posts (89.4%) were posted from accounts that communicate primarily about e-cigarettes. The e-cigarette posters were more active on the platform in terms of both content publication (posting) and networking (number of followers and following). These results suggest that efforts to control e-cigarette/cigarette content on Instagram should take multiple approaches. For e-cigarettes in particular, in the context of Instagram, the priority would be to exert control over e-cigarette content by regulating marketing from e-cigarette businesses through their official accounts, employees’ accounts, and sponsorships. Both cigarettes and e-cigarettes require the surveillance of the activities of communities and enthusiasts on Instagram [32]. Lastly, as many people post cigarette or e-cigarette content as part of their everyday lives, emphasis should be placed on designing and implementing interventions with the goal of changing people’s perceptions of sharing this kind of content on social media beyond the simple consumption of cigarettes and e-cigarettes.

Further, we identified some notable differences between the cigarette posts and e-cigarette posts in terms of their respective content. The majority of e-cigarette posts were presumed to have a marketing intent (56.3%), and 13.8% of the e-cigarette posts were found to share specific promotional information. In line with the results, a large portion of the e-cigarette posts portrayed a brand in the photograph/video content (63.0% of e-cigarette posts as compared to 15.8% of cigarette posts). The results clearly indicate a need to restrict e-cigarette marketing practices on social media. Instagram users spent 11.2 h on the mobile app per month in 2021 globally [43]. Exposure to brands on the platform may influence awareness of brands and further consideration and purchase of those brands. Exposure to a brand on Instagram can easily lead to searches for further information and to purchases.

On the other hand, portrayals of daily life (73.2% vs. 41.3%) and portrayals of humans (80.3% vs. 43.7%) were more frequent in photograph/video content shared in cigarette posts. Likewise, posts about cigarettes were three times as likely to present smoking than posts about e-cigarettes were to present vaping (67.1% vs. 21.3%). These results show that cigarette posts tend to portray the act of smoking in the context of everyday life, whereas e-cigarette posts tend to focus on a brand. The prevalence of portrayals of smoking/vaping on Instagram is problematic given that such portrayals have the potential to model behavior for the platform’s users as suggested by social cognitive theory [39,44]. Compared with product images, everyday posting of smoking/vaping behavior on the part of laypeople may have a stronger effect in terms of modeling behavior. In line with this observation, researchers have already raised concerns over the potential of social media to normalize the use of both cigarettes and e-cigarettes [32,45]. Thus, even without marketing practices, continuing surveillance of cigarette and e-cigarette content on social media is crucial, and effort is needed to change people’s attitudes towards sharing smoking/vaping content on social media. Moreover, the depiction of smoking/vaping lifestyle overlaps with the tobacco industry’s practices to advertise under the radar through various media including movies and music videos [46,47]. It would be important to monitor and regulate the tobacco industry’s potential use of seemingly everyday posts to normalize cigarette or e-cigarette consumption.

Furthermore, we examined the nature and extent of user engagement with the posts. Social media allows users to engage with posts through features such as “liking” and “commenting”. Yang and colleagues indicated a positive association between young adults’ engagement with ENDS-related content posted on social media and subsequent ENDS use [48]. In the present study, we did not find a significant difference in the number of likes accruing to e-cigarette posts and cigarette posts, although the former attracted more comments. However, we do not have sufficient information to interpret the difference. More importantly, the user engagement features stress another reason why it is important to regulate cigarette and e-cigarette content on social media: Active interaction with such content may increase the likelihood of future cigarette/e-cigarette use [48].

One potential limitation of the present study relates to the sampling method used. We identified e-cigarette posts and cigarette posts through the hashtag search described. Based on this approach, only posts with the designated hashtags were included in the sample, which may have led to bias in the results. For example, laypeople who post about their daily lives may not be concerned with adding hashtags related to cigarettes/e-cigarettes, whereas companies and industry people can be expected to ensure that they use given hashtags in order to make their posts searchable. The potential underrepresentation or overrepresentation of certain types of posts should, therefore, be considered in contextualizing the results reported.

In addition, the present study used only four general hashtags to sample posts for each kind of tobacco product. While the use of general search terms allowed us to sample cigarette posts and e-cigarette posts in a relatively equivalent manner, again, there is the limitation of missing out cigarette/e-cigarette posts without the designated hashtags. Among the posts without designated hashtags, there can be posts including the hashtags more specific to smoking/vaping activities or culture such as “cloudchasing” for e-cigarettes. A more comprehensive investigation of the hashtags used for cigarette and e-cigarette posts would help achieve a more representative sample of the posts for future research.

Further, while the present study compared e-cigarette posts and cigarette posts along with various aspects, there are still numerous patterns that were not captured in the study. Especially, the photographs/videos shared in the posts convey ample information. Future studies need to conduct a more in-depth comparison possibly reflecting more nuanced message contents such as humor, violence, emotions, and sexual connotations [33]. Moreover, experimental studies are needed to better understand the effects of the portrayals in the photographs/videos on the viewers’ engagements, perceptions, and subsequent cigarette/e-cigarette consumption.

Lastly, the present study is also limited in that we investigated only one social media platform, Instagram. The results, therefore, reflect the characteristics of Instagram and may not be generalizable to e-cigarette and cigarette posts on other platforms. In light of the prevalence of e-cigarette use among youth, a critical direction for future research would be to investigate other social media platforms popular among youth such as TikTok and Snapchat [29]. TikTok, in particular, has emerged as another key platform for marketing e-cigarettes [49], which means there is a pressing need to understand and regulate e-cigarette marketing in that context.

## 5. Conclusions

In conclusion, we analyzed the content of e-cigarette posts and cigarette posts on Instagram. The comparison highlighted differences between e-cigarette posts and cigarette posts in terms of sources, content, and engagement. The findings broaden the field’s understanding of cigarette and e-cigarette content on Instagram with implications for monitoring and regulating that content in the interest of public health, especially in regard to younger populations.

## Figures and Tables

**Table 1:** Ratio of posts by source type.

Posts by	E-Cigarette	Cigarette
Layperson	27.2%	76.8%
Industry person	18.5%	0%
Cigarette/e-cigarette company	40.9%	1.3%
Health organization/practitioner	0%	0.4%
Community	13.0%	19.3%
Other/unidentifiable	0.4%	2.2%

**Table 2:** Classification of sources.

User Account	E-Cigarette	Cigarette
Layperson	27.8%	86.8%
Industry person	19.8%	0%
Cigarette/e-cigarette company	41.8%	0.5%
Health organization/practitioner	0%	0.5%
Community	10.1%	9.9%
Other/unidentifiable	0.4%	2.6%

**Table 3:** Account activities according to source type (mean (standard deviation) and median).

Source (User Account)	Number of Posts	Number of Followers	Number of Followings
Layperson	364.97 (600.26) 132.00	1585.99 (7309.01) 321.00	554.96 (1066.87) 258.50
Industry person	662.70 (901.24) 322.00	11,365.79 (52,578.59) 1182.00	1412.62 (1704.84) 854.00
Cigarette/e-cigarette company	1067.53 (2037.70) 311.50	21,136.45 (54,279.17) 3655.50	1336.40 (1819.94) 696.00
Health organization/practitioner	312.00 (NA *)	2361.00 (NA *)	2411.00 (NA *)
Community	786.09 (1103.53) 267.00	30,915.06 (54,122.82) 2461.00	786.81 (1338.76) 206.00
Other/unidentifiable	1559.00 (2654.40) 584.50	18,728.67 (17,687.35) 18,750.00	2478.00 (3092.01) 1068.00

* Only one case of health organization/practitioner.

## Data Availability

The data presented in this study are available on request from the corresponding author.
